# A Scalable and Modular Dome Illumination System for Scientific Microphotography on a Budget

**DOI:** 10.1371/journal.pone.0153426

**Published:** 2016-05-03

**Authors:** Ricardo Kawada, Matthew L. Buffington

**Affiliations:** 1 Programa de Pós-Graduação em Entomologia e Conservação da Biodiversidade, Universidade Federal da Grande Dourados, Rodovia Dourados-Itahum, Km 12, Cidade Universitária, CEP 79804–970, Dourados-MS, Brazil; 2 Systematic Entomology Laboratory, ARS-USDA, c/o National Museum of Natural History, Smithsonian Institution, 10th & Constitution Ave NW, MRC 168, PO Box 30712, Washington, DC 20013, United States of America; University of Cambridge, UNITED KINGDOM

## Abstract

A scalable and modular LED illumination dome for microscopic scientific photography is described and illustrated, and methods for constructing such a dome are detailed. Dome illumination for insect specimens has become standard practice across the field of insect systematics, but many dome designs remain expensive and inflexible with respect to new LED technology. Further, a one-size-fits-all dome cannot accommodate the large breadth of insect size encountered in nature, forcing the photographer to adapt, in some cases, to a less than ideal dome design. The dome described here is scalable, as it is based on a isodecahedron, and the template for the dome is available as a downloaded file from the internet that can be printed on any printer, on the photographer’s choice of media. As a result, a photographer can afford, using this design, to produce a series of domes of various sizes and materials, and LED ring lights of various sizes and color temperatures, depending on the need.

## Introduction

Lighting is the language of photography. Patterns of light convey information just as surely as spoken words. The effective size and direction of the light source to the subject is one of the most important relationships in photography. It determines what types of shadows are produced and may affect the type of reflection. Light can reflect from a subject as diffuse reflection, direct reflection, or glare. Most surfaces cause some of each of these three types. The proportions of each type of reflection vary with the subject, and it is the proportion of each reflection in the mix that makes one surface look different from another. Understanding the basic principles of photography in the current age of digital imaging is critical in deciding what lights are needed, and where, before we begin to place them. Once this is established, the rest is just fine-tuning [1].).

Images have always been of fundamental importance in taxonomy and for the documentation of natural history specimens. In recent years, however, digital photography and other imaging techniques are rapidly transforming the way in which we take and portray images in these fields. Part and parcel to these developments has been a push to improve the lighting of insect specimens for such photography, especially in circumstances where very high magnification (<40x) is needed to ‘fill the frame’ of a typical digital camera. [2] pointed out that ‘naked’ light, under high-magnification conditions, produces glare and flaring at such an alarming rate as to actually obscure structural details of interest to the researcher. The conclusion of [2] was to develop intense, yet diffuse, lighting techniques that provide enough photonic energy to effectively illuminate the subject, while at the same time, disperse the photons such that the subject is within a photonic cloud of purely indirect, and therefore scattered, light.

Some techniques currently employed to create soft yet intense light include projecting halogen fiber optic lights through diffusers such as Mylar, passing light over rough Styrofoam edges, or constructing lighting chambers from Styrofoam [2, 3]. However, these methods typically involve a great deal of setup time and experience.

Another widespread technique, that has been shown to be both simple and effective, is dome lighting [4,5]. This technique uses soft, white light diffused from the inside of hemispherical domes placed above light sources (typically LED ring lights). Both authors have experimented with a variety of light box forms to create diffuse lighting for insect imaging, and have found the best light arena is one created within a hemispherical dome [4].). Though commercially available illumination domes exist, they can be expensive, while creating a dome lighting system of your own is in fact easy and inexpensive (see [4]). Here we expand on [4] and [5] by providing an entirely scalable dome system, where the user/builder can custom design the precise size dome they need for the application at hand. We envision a photographer having 3–5 domes of various sizes for various applications, all based on the single dome and ring-light design presented here. Additionally, the components of this system are easy to obtain, affordable, and construction of the dome is a relatively simple and rewarding experience. Further, the materials used to make the dome and the LED ring lights can be varied according to the users’ needs, and experimentation with various building materials is strongly encouraged by the authors.

The construction of the two fundamentally different dome designs explained here are based on a large dome used in conjunction with a ‘zoom-type’ microscope system (e.g. the Leica M10 lens) (described by [4]), and a mini-dome used in conjunction with a compound microscope (described by [5]). The large dome system is ideal for large insect specimens, as the working distance of zoom lenses allow for the entire dome to be inserted under the objective lens, and these larger domes are needed for large specimens. The mini-dome system is for insect specimens 5mm or smaller in size, where the resolving power of the zoom lens is not sufficient to render smaller details. The advantages and disadvantages both systems, as well as suggested equipment, are discussed in [4] and [5],), both of which are part of an ‘instant symposium’ devoted to insect illustration in *American Entomologist*.

The dome design utilized in this study is nearly 100 years old. Walther Bauersfeld (Carl Zeiss Company) required an efficient dome for a planetarium projector designed by him in the 1920’s. Bauersfeld’s very light and very strong dome was patented and installed on top of the Zeiss plant in Germany. Buckminster Fuller, some years later, dubbed this dome design a *geodesic dome*, and patented the mathematical principles behind the geodesic dome in the 1950’s; his goal was to provide designs for the full-scale construction of affordable housing at reduced costs using this design. While that goal was never fully realized (for various reasons), some truly spectacular and bizarre buildings were designed using the technique, including *Spaceship Earth* at Epcot Center in Orlando, Florida, and the *Cinerama* in Hollywood, California.

The geodesic dome design is appealing here as a lighting dome for the same reasons the design is well-suited for construction: the dome is actually a complicated set of interlocking flat panels, all with straight edges, which are easily cut and the whole set fabricated together. Additionally, since all of the panels conform to single set of mathematical principles (the core of which is the icosahedron), the size of any dome is easily scalable, and the base diameter is always in proportion to the volume of the space enclosed by the dome. Both of these attributes are critical in the success of the dome presented here: construction is a simple matter of gluing together panels of triangles and pentagons, and sizing the dome to each person’s need comes from a scaling function of the computer printout of the design. We hope this design will be bring effective photographic illumination to a broader community of research entomologists, and enthusiasts in general, thereby improving the overall quality of insect photography and illustration worldwide.

## Material and Methods

Specimens were sorted from ethanol and critical point dried (CPD); specimens were then adhered to either black or white point mounts with adhesive. The specimens were photographed under a Leica Z16 APO stereomicroscope and Leica DM2500 microscope fitted with a camera adaptor coupled to a Leica DFC 295 video camera (Leica Microsystems, Switzerland); the program Leica LAS (Leica Application Suite V3. 6.0; Microssystems by Leica (Switzerland) Limited) was used to capture individual focal planes. The LAS settings used to generate the images from the dome described herein were: exposure, 166.2ms; gain, 1.0x; saturation, 1.2; gamma, 0.60; for the Leica dome, we used: exposure, 400.1ms; gain, 1.0x; saturation, 1.2; gamma, 0.60. The equipment used for data storage was a high-performance notebook with Windows 7 Professional operating system and an Intel (R) Xeon (R) central processing unit (CPU). Helicon Focus (HeliconSoft) software was responsible for stacking the layers into a single combined-focus image using the following parameters: method C and full resolution. No further manipulation or enhancement was performed on the images presented here. The commercially available dome used here for comparison was the Leica LED5000 HDI.

**Fig 1:**
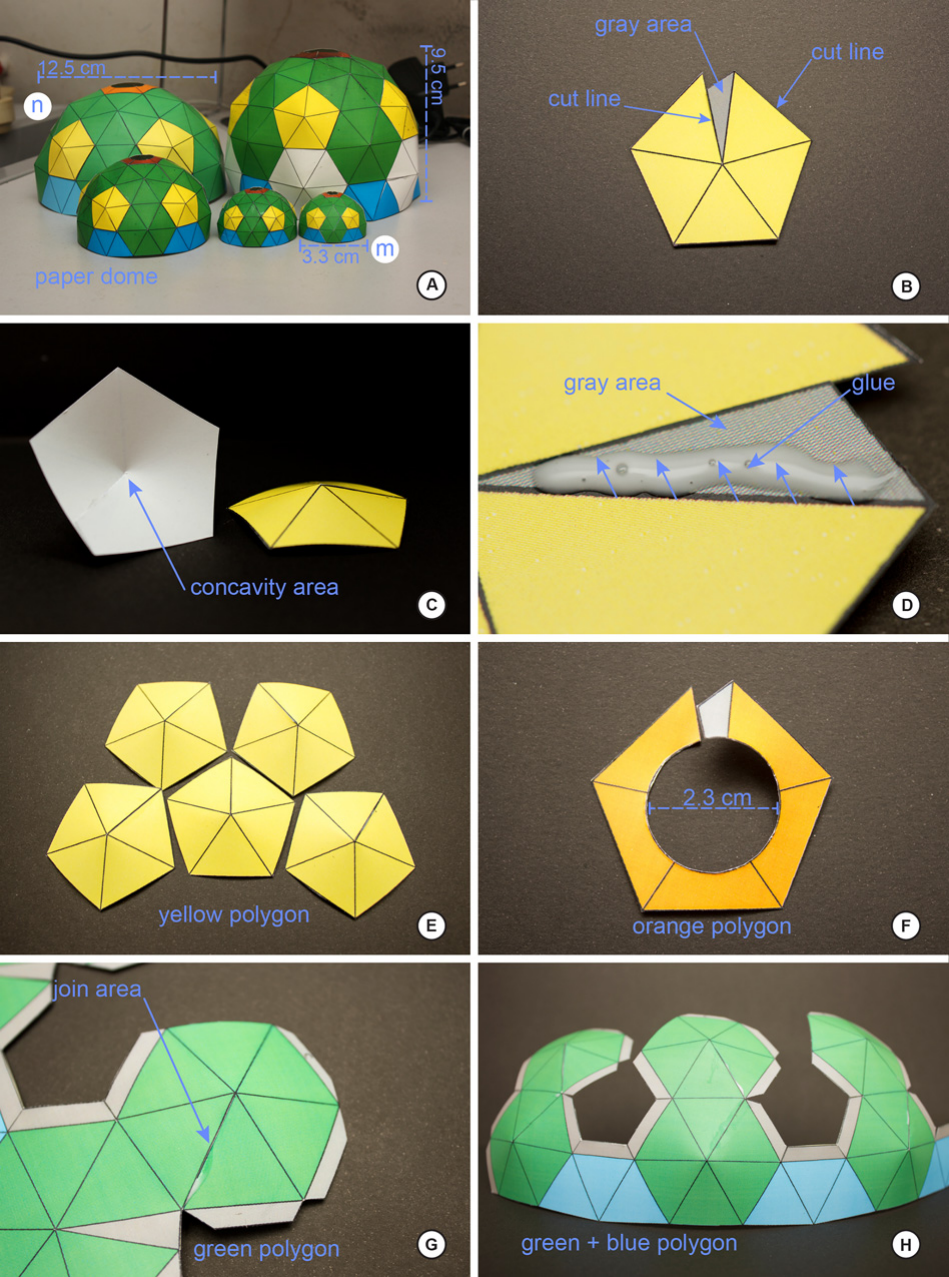
Step-by-step of construction of the standard size dome, part 1. A, the variety of dome sizes that can be produced from the S1 Appendix template; B, detail of the pentagonal segment of the dome; C, how the pentagonal segment should be curved following cutting and gluing; D, detail of area to apply glue in the pentagonal segment; E, the five pentagonal segments ready for application to the dome frame; F, the oculus pentagonal segment the comprises the top of the dome; G, detail of the green hexagonal segments, indicating the glue point for introducing the interior curvature; H, the base of the dome taking shape.

**Fig 2:**
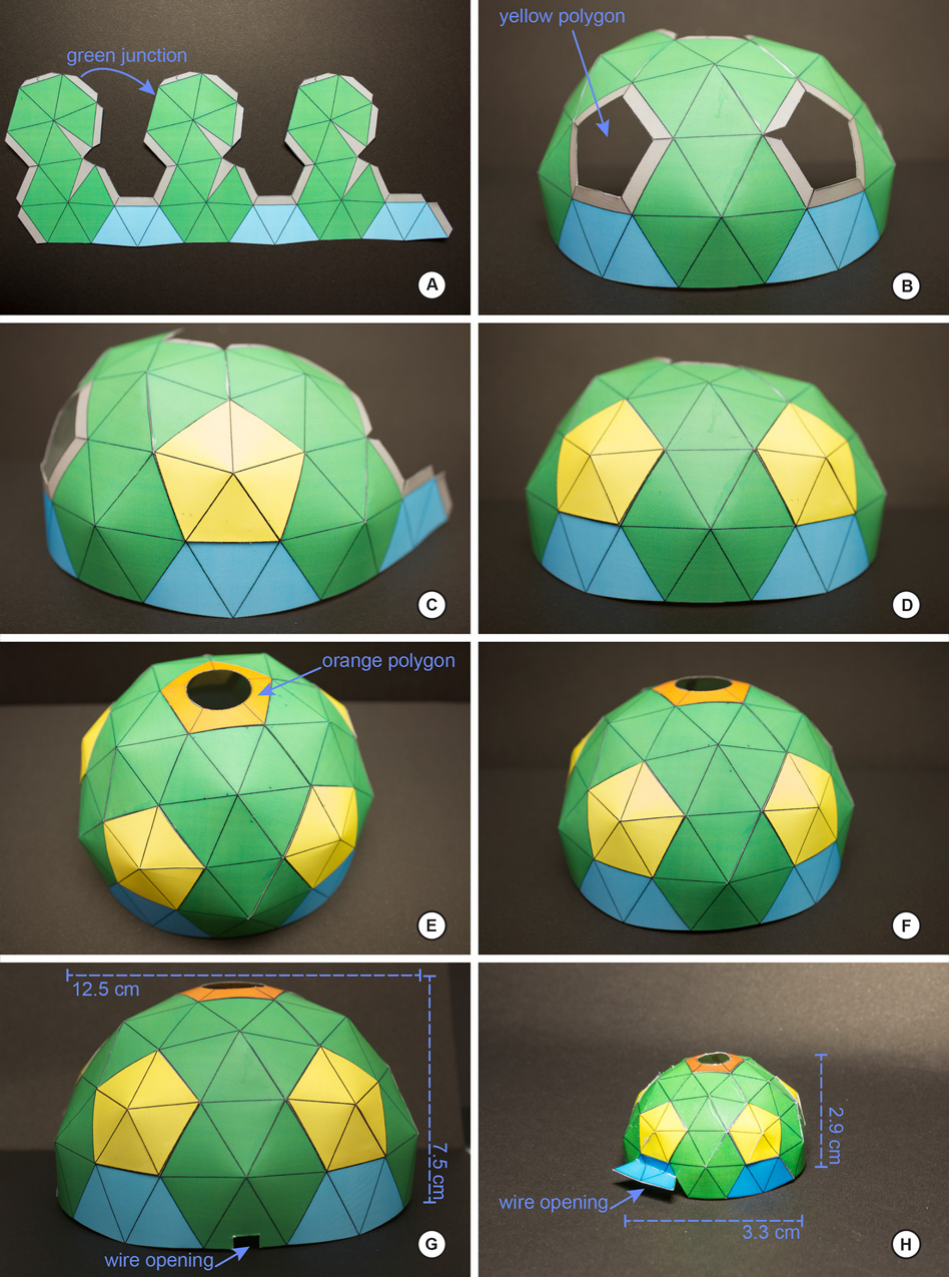
Step-by-step of construction of the dome, part 2. A, detail of the hexagonal segments; arrow indicates the glue points between segments (gray areas); B, the basic frame of the dome is ready for the pentagonal segments to be applied; C, a correctly installed pentagonal segment; D, the completed dome taking shape; E, the correctly installed oculus pentagonal segment; F, the completed dome; G, dimensions of the completed dome as constructed using the default template size; note the notch for the LED ring power cord; H, the completed mini-dome (with dimensions), with a relatively larger wire opening for the LED ring power cord.

**Fig 3:**
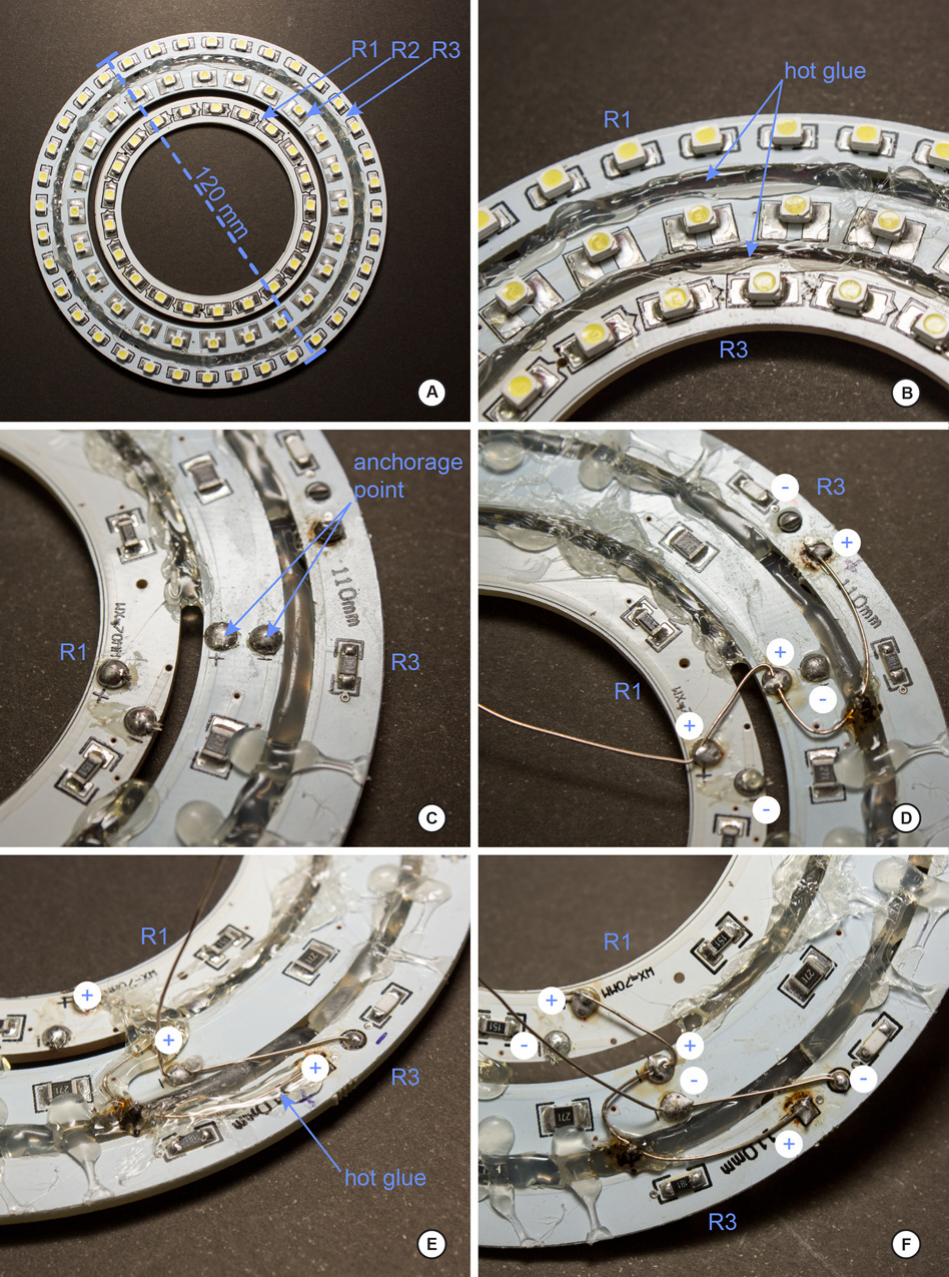
Step-by-step of construction of the LED ring light, part 1. A, for the default large dome size, the three LED ring light should abut one another, with a resulting outer dimension of 120mm; B, detail of how the LED rings are joined; C, detail of the alignment of the power points of the individual rings (anchorage points); D, connection, in series of the positive power lead; E, detail of using glue as an insulator between the positive and negative leads; the completed positive and negative connections; note, the insulating glue layer has not yet been applied here.

**Fig 4:**
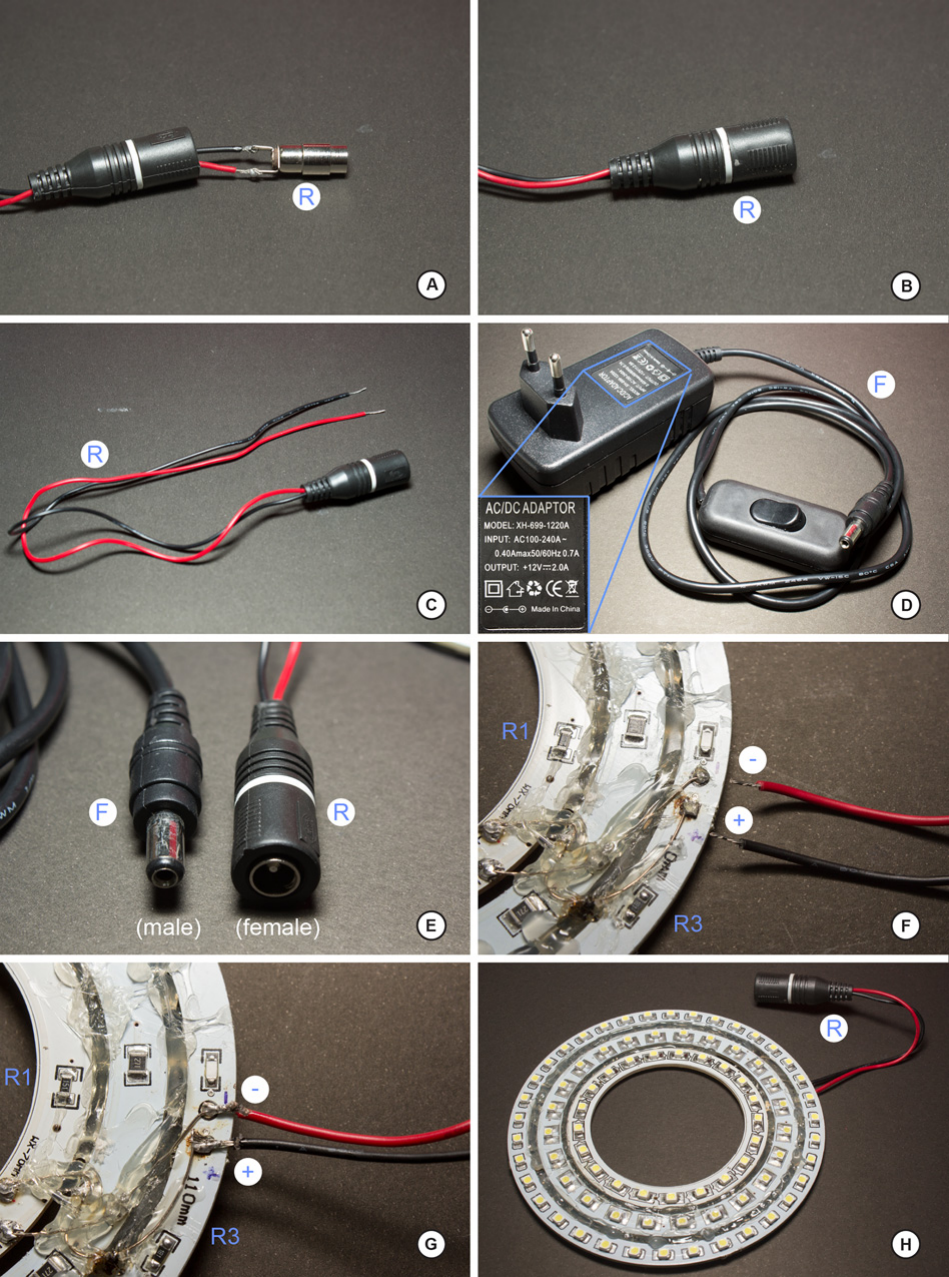
Step-by-step of construction of the LED ring light, part 2. A, connector R soldered to the positive and negative leads that go to the ring lights; note the yoke that encompasses the soldered connector must be threaded onto the leads prior to soldering connector R; B, after soldering, connector R should reside within the yoke; C, the complete connector R and positive/negative leads, ready for soldering to the LED ring lights; D, converter that powers the LED ring light; note the inset describes the specifications of the adaptor as well as the polarity of connector F; this is critical for the correct wiring of connector R; E, close-up of the two connectors F and R; F, detail of the connection of the positive/negative leads to their respective poles on the LED ring light; G, correctly soldered leads to the LED ring light; H, the completed LED ring light should resemble the ring light figured here.

**Fig 5:**
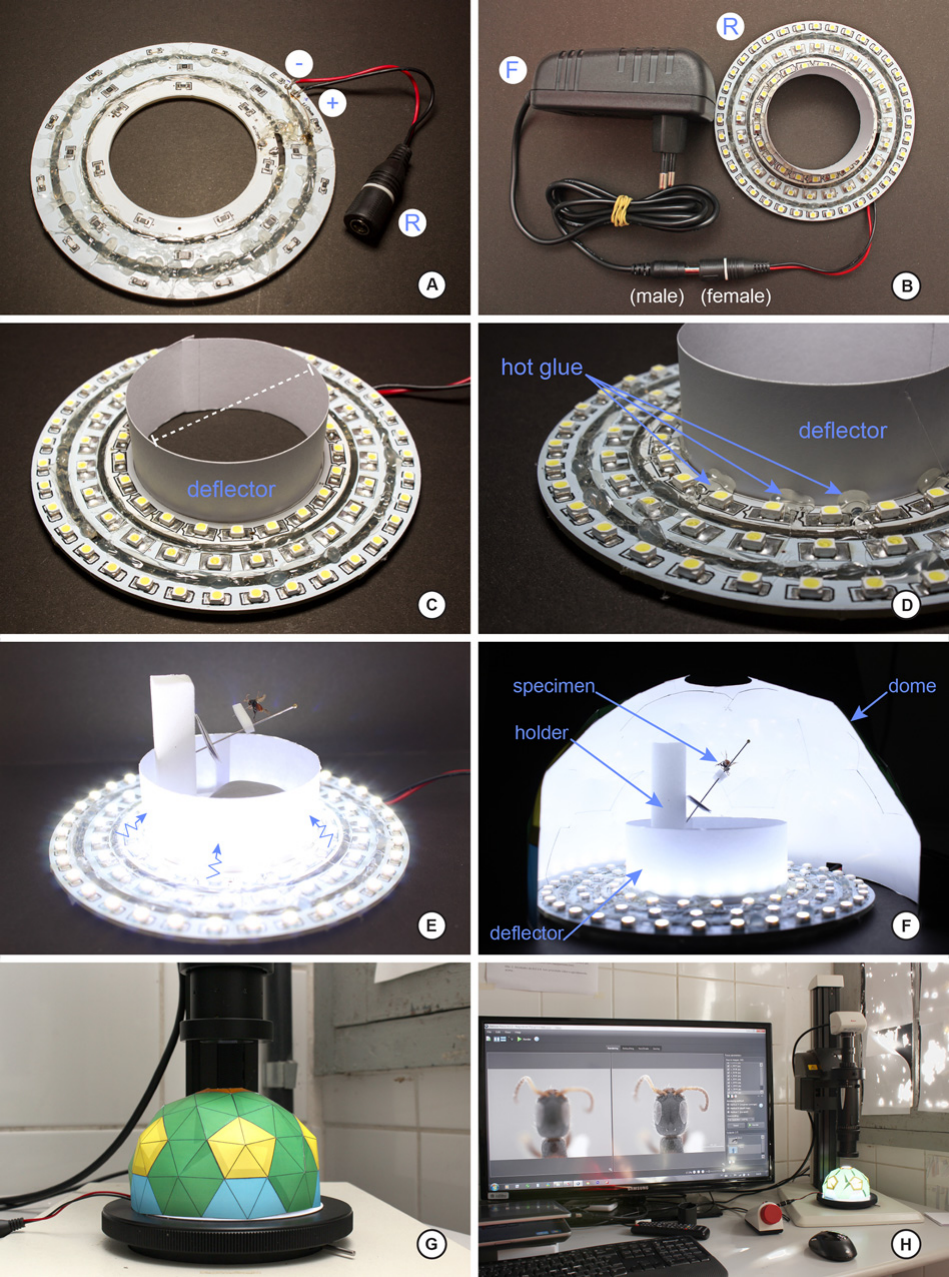
Step-by-step of construction of the LED ring light, part 3. A, ventral view of the LED ring light and connector R awaiting connection to F; B, the completed ring light with power adaptor connected, ready for work; C, overview of the internal deflector ring needed to disperse the LED light to the interior of the dome; D, suggested gluing points along the deflector ring; once glued, the deflector ring doubles as a handy grip for moving the dome; E, illuminated LED ring lights, illustrating the function of the deflector ring; note a suggested specimen holder made of foam within the ring; F, cutaway of the entire dome setup in action; G, overview of the illumination dome under a zoom lens setup; note the working distance of this lens allows for the dome to be safely located under the lens within the lens hitting the dome; H, the completed setup at work, utilizing external equipment described in Materials and Methods.

**Fig 6:**
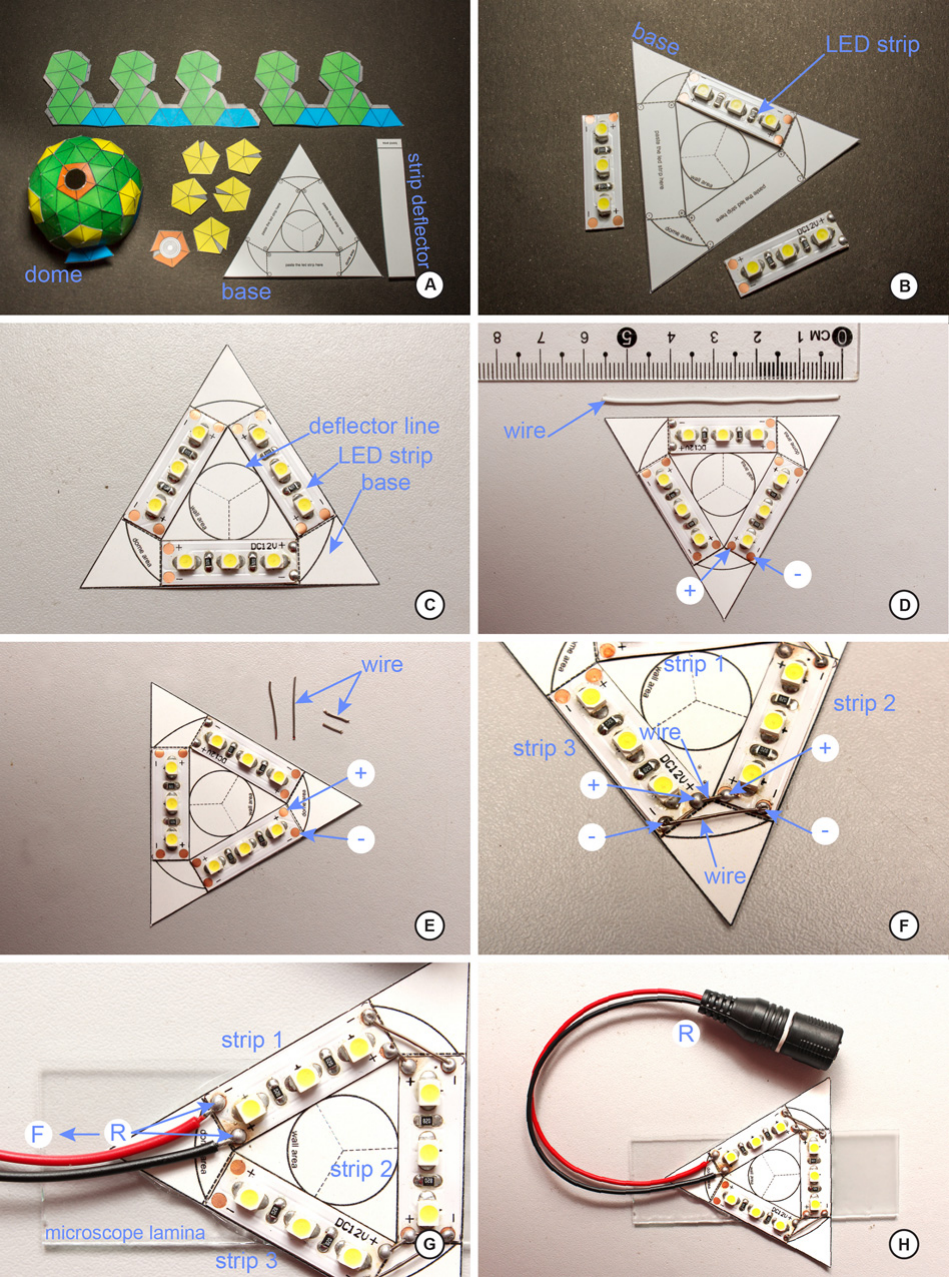
Step-by-step of construction of the mini-ring, part 1. A, the complete set of templates needed; while the dome itself is a scaled-down version of the dome in S1 Appendix, the LED triangle base is an additional template to cut-out; B, adhere the LED strips at the points suggested on the template; C, detail of the LED triangle and the location of where various parts of the dome are to be located; D, preparing the LED triangle for wiring; note the scale of not only the triangle, but the length of wire needed; E, wire prepared for soldering into position; F, detail of connectors soldered into place; note the location of the positive/negative poles; G, connection of the power leads to the LED triangle; as in the larger LED ring light, the LED strips in the triangle are wired in series; H, the completed LED triangle ready for connection to the power adaptor.

**Fig 7:**
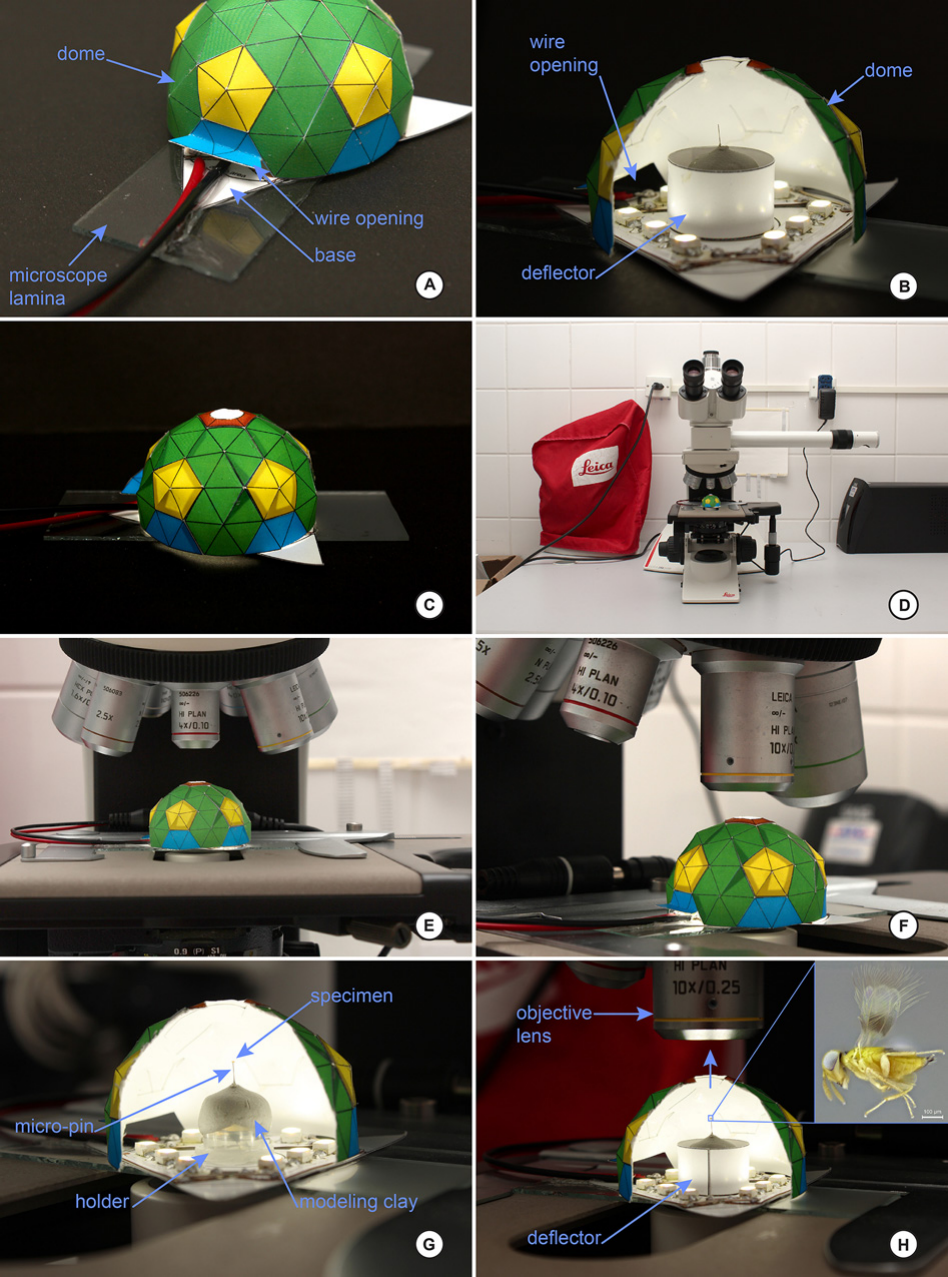
Step-by-step of construction of the mini-ring and the mini-dome in action. A, the completed mini-dome with LED triangle illumination stage; note the triangle is mounted on a microscope slide (for interfacing with the compound microscope stage); also note the small door providing egress of the power leads; B, cutaway of the mini-dome in action; C, close-up of the mini-dome in action; D, the mini-dome installed on a Leica compound microscope; E and F, close-up illustrating the orientation of the mini-dome to the compound lens; note the dome must be small enough to fit under the lens without the lens hitting the dome; G and H, cutaway illustrating the suggested mounting technique for micro-insects while using the mini-dome (from Buffington and Gates 2008); insect is an example image of an aphelinid wasp (Chalcidoidea) whose total body length is ca. 0.5mm.

**Fig 8:**
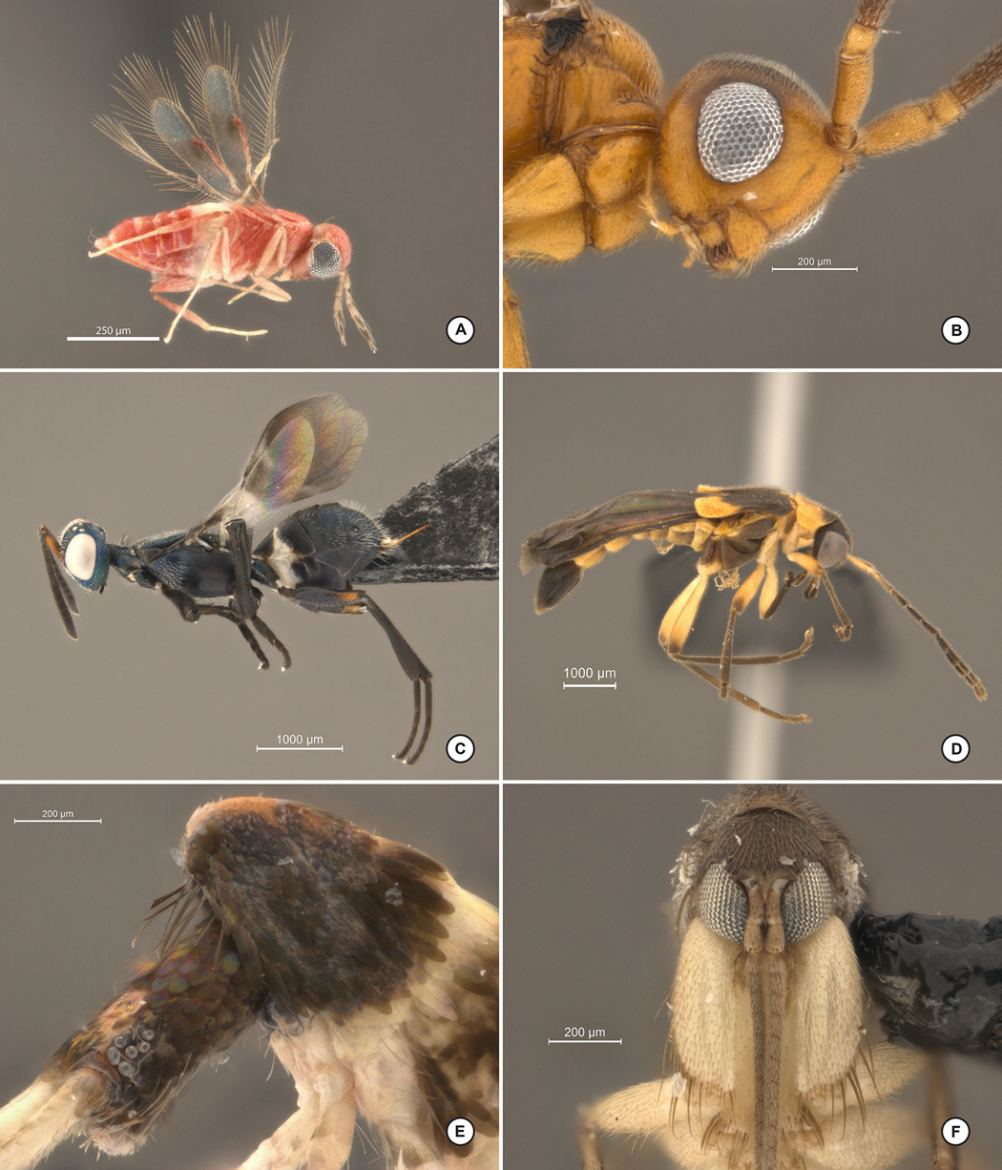
Images capture on stereomicroscope using dome and LED ring light. A, Hymenoptera: Aphelinidae in lateral view; B, Hymenoptera: Embolemidae detail of head and pronotum in lateral view; C, Hymenoptera: Eupelmidae in lateral habitus; D, Coleoptera: Cantharidae: *Malthoichthyurus* sp. habitus in lateral view; E, Collembola: Entomobryidae head and thorax in lateral view; F, Diptera: Mycetophilidae head in frontal view.

**Fig 9:**
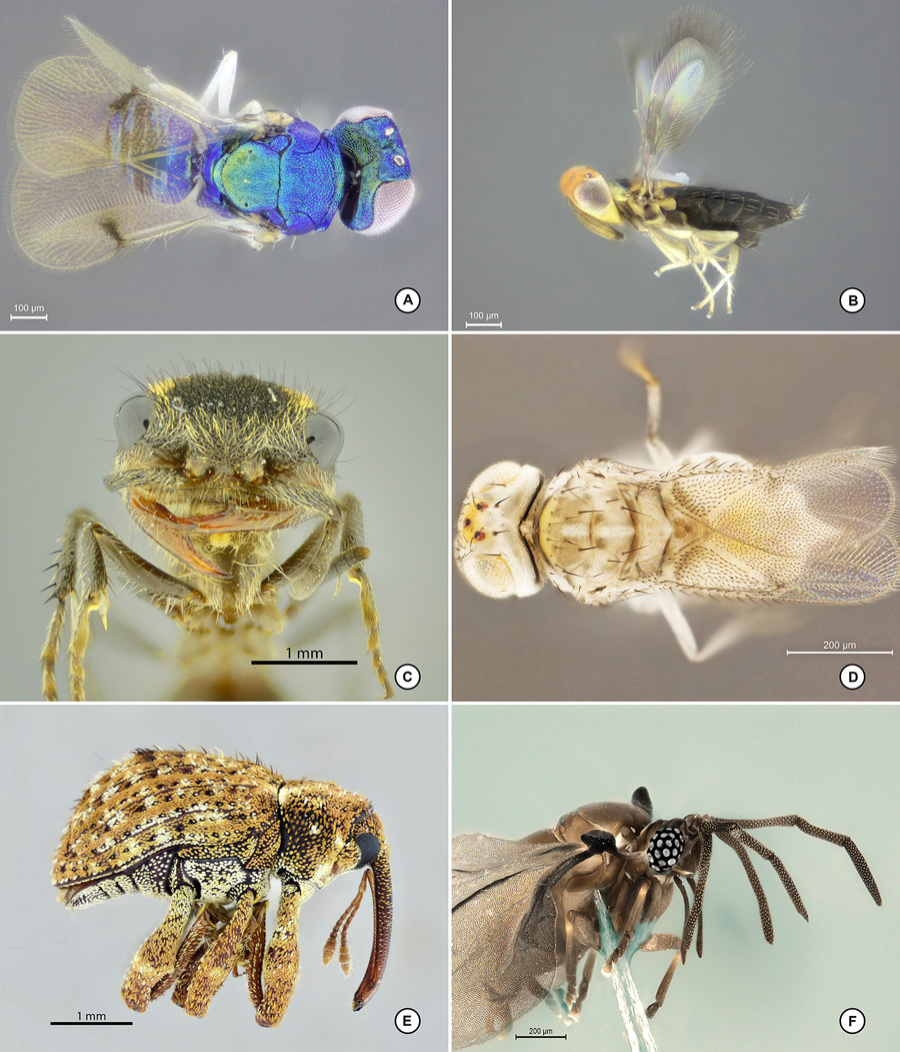
Images capture using a compound microscope, the mini-dome and mini-ring. A, Hymenoptera: Eulophidae in dorsal view; B, Hymenoptera: Signiphoridae: *Signiphora* sp. in lateral habitus; C, Hymenoptera: Mutillidae: *Pertyella* sp. head in lateral view; D, Hymenoptera: Eulophidae habitus in dorsal view; E, Coleoptera: Curculionidae: *Conotrachelus* sp. habitus in lateral view; F, Strepsiptera head and thorax in latero-frontal view.

**Fig 10:**
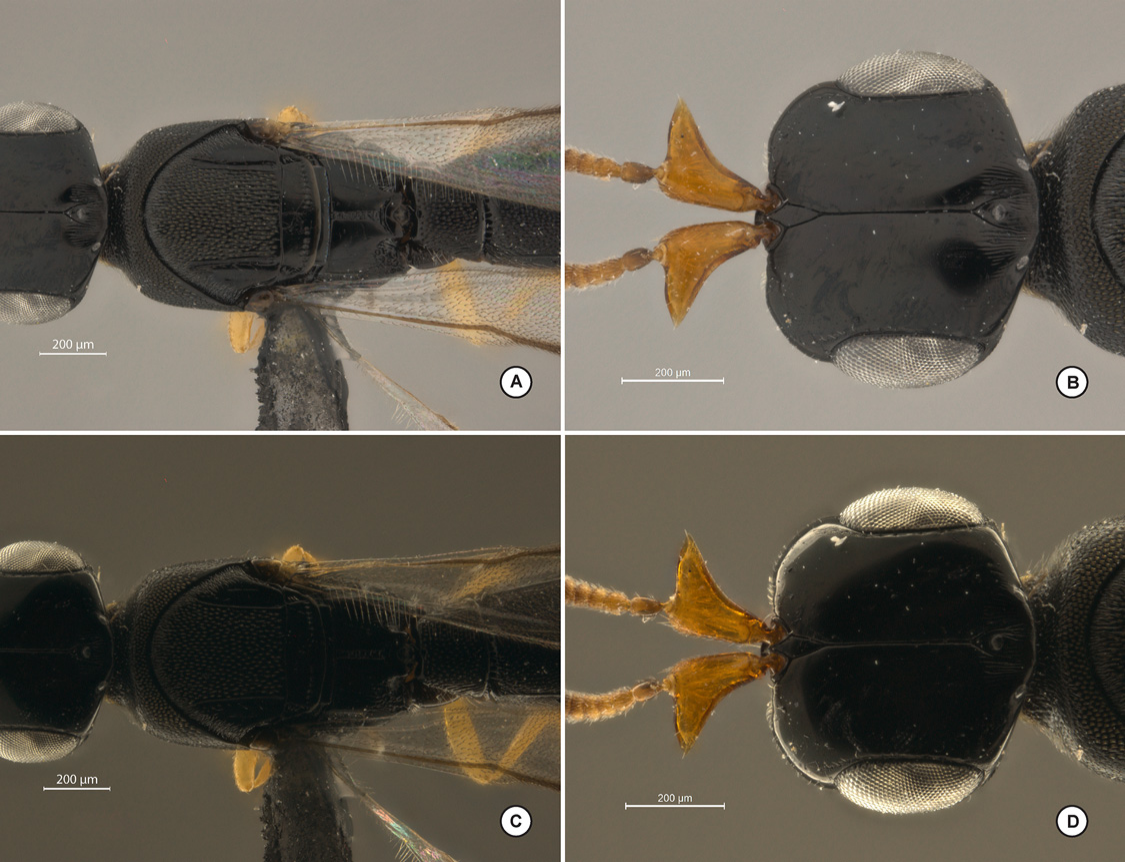
Comparison of the dome system described herein (A-B) and the commercially available dome system from Leica (C-D). Taxon: Hymenoptera: Platygastridae.

Below we explain the procedure for the construction of the dome and two different LED ring lights as built for this study. Figs 1 and 2 illustrate the various stages and shapes of the dome. Figs 3 and 4 illustrate the construction of the LED ring lights. Fig 5 shows the completed dome. Fig 6 illustrates the construction of the mini dome. Fig 7 shows the both dome designs in use. Figs 8 and 9 demonstrate the wide range of insect taxa that can be imaged using this affordable dome system. Fig 10 compares the results obtained with the dome described here, with commercially available lighting dome from Leica. All materials used, as well as sources, are described in S3 Appendix.

## Results

Building the Dome (Figs 1–3). The procedure for the construction of the dome lighting is described by the sequence below:

1) Print the dome light (S1 or S2 Appendices) on a sheet of cardstock, preferably A4 size, and minimum weight of 120 lbs/square inch. When printing through Adobe Photoshop CS, be sure to scale to fit media. Also note that scaling at this stage can be used to generate domes of various sizes for different applications (Fig 1A). We advise when building your first dome that you print the template on a color printer, as the colors are used to denote precise gluing faces, as well as different colors indicate the order in which different parts of the dome require construction.
2) All polygons marked with outline and dotted line can be cut with scissors or hobby knife.
3) Once the template is cut out, the next step is the process of joining the pieces with a glue stick or hot glue-gun. However, it is important to note that the inner lines of each polygon *do not* need to be folded or cut. Each polygon, individually, was designed to make the angle necessary to generate the stiffness needed of the dome when fully assembled.
4) Assembly of the **yellow polygon** (5 pentagons total): place adhesive in the **gray areas** (Fig 1B and 1D) of the **yellow polygon** and wait at least 10 seconds to join the sides. Once joined, hold for at least 10 seconds for the glue properly set. Repeat for all five polygons (Fig 1E); the completed polygons will have a natural concavity (Fig 1C).
5) Preparing the **orange polygon** (1 hexagon) is similar as the steps for the yellow polygons; place glue on the grey area and join, holding until the glue sets. This piece will form the oculus for the light path to the camera, so care should be taken to cut the center circle very carefully.
6) Preparing the **green polygon** (5 pairs with 10 hexagons). **Template Part 1**: place glue on the narrow, triangular sections and join together (labeled ‘join’, with arrows, in the template, S1 Appendix). The gluing of these areas results in the concavity needed to give shape to the dome, and once these glued areas are dried, the base of the dome should be taking shape (Fig 1H). Next, join the gray tab labeled ‘a’ to the opposite lobe, following the path of the arrow on the template; repeat this step for joining the middle lobe with the last lobe on the right. The dome will now begin to take shape (Fig 1H).
7) **Template Part 2:** This section is simply **Part 1** but with only two lobes and not three; follow the same instructions for the assembly of **Part 1** here for **Part 2** by joining the grey tab on the first lobe to the receiving end on the second lobe.
8) Once **Part 1** and **Part 2** are joined and dried, the final step will bring the dome closer to completion by joining the bases of **Part 1** and **Part 2** together; achieve this by gluing **tab b2** of **Part 1** to the corresponding **b2** receiver on **Part 2**; glue **tab b1** of **Part 1** to the corresponding **b1** receiver on **Part 2**. The result should resemble the dome in Fig 2D.
7) Next, glue the yellow polygons from Step 4 to the grey areas surrounding the pentagonal holes in Fig 2B; the result should resemble Fig 2D.
8) Joining the **orange polygon** (Step 5) on to the top of the dome on the gray tabs (Fig 2E); the dome should resemble the dome pictured in Fig 2F.

NOTE. Construction of domes, using the template of S1 or S2 Appendices, all follow the same construction method. If different sizes of domes are desired, simply adjusting the printing size of the template will achieve the desired outcome of sizes.

Building the LED ring light (Figs 3–5). The procedure for the construction of the ring lighting is described by the sequence below; if different diameters are required for different sizes of domes constructed in the previous section, simply obtain LED strips of various curvatures as needed. In the example presented here, we used UxCell LED rings of 80/100/120mm diameters embedding 24/33/39 SMD white LED (colour temperature 6000K); these were purchased from Amazon (manufacturer part numbers a11030700ux0452, a14012000ux0488 and a14012000ux0491, respectively). The in-series wiring is the same regardless of diameter. All other materials needed are summarized in S1 Appendix.

1) The first step is to unify the LED rings to become a single ring with three rings of light (Fig 3A), each ring a different diameter.
2) Join the three LED rings (Fig 3A) named as **R1** (inner), **R2** (middle) and **R3** (outer) of different diameter with hot glue (Fig 3B) to sturdy base material. If using the default dome template size as found in S1 Appendix, the outer ring **R3** must have no more than a 120 mm diameter (Fig 3A; if a larger dome template is printed, then a larger LED ring light assembly is warranted; if a ‘mini-dome’ is required, a smaller, triangular LED light setup is needed (next section).

The next step is the connection of the wires on the LED rings, which will power the LEDs.

3) Each LED ring has two connection points (+/-, Fig 3C); with three LED rings, we have a total of six points (3+/3-; Fig 3D) to connect; these concentric rings of LED strips will be wired in-series. Connecting the wires requires a bit of dexterity, and experience with soldering electrical wires helps. To facilitate the process of soldering, put a small amount of solder paste on each connection point (Fig 3C). The solder paste will facilitate the connection of the wires.
4) Solder the connection points of the positive poles (Fig 3D) for each LED ring, and then cover with hot glue (Fig 3E). The adhesive here has the dual role of both strengthening the connection, as well as insulating the connection (as positive/negative connections are very close here (Fig 3F).
5) Repeat step **4)** for each of the negative poles, resulting in something resembling Fig 3F).
6) Having completed steps **4)** and **5)**, the LED ring is ready to receive the power (Figs 4 and 5).
7) Solder the **R** connector (Fig 5A) to the wire that is attached to the LED rings (Fig 4F & 4G). This connector will serve as an intermediary between the LED ring and the source (**F)** of the 12 volt power supply (Fig 4D). If you have dome lights of various sizes, only a single 12 volt power supply will be needed for each dome light system to plug into. Here, the R connector is considered the "male" end of the connection and the source of 12 volts is the "female" connector (Fig 4E).
8) It is essential to have the positive and negative wires (typically indicated by different colors of insulation) of the LED rings to correspond to the correct terminal posts of the R and F adaptors. In our case, the positive pole is black and red pole is negative (Fig 4G). Verify the polarity of the power supply (F) by examining the wall-mount transformer, such as the one in Fig 4D; note in the bottom of this figure (inset), the polarity of the female end of this plug is pictured.
9) Make the solder of the plug connector **R** to the outer ring R3 (Fig 4F & 4G); since the rings of LEDs are connected in-series, only one connection point is needed to the power source. The solder point should be on the ventral surface of rings (Fig 4G & 4H; Fig 5A). Once plug R is inserted into receiver F (Fig 5B), the LED ring light should be functional.

The next step is to cut the cardboard ring that prevents direct LED light from directly hitting the specimen being photographed.

10) Cut a strip of cardboard of about 20 mm wide and long enough to cover the smallest diameter (R1) of the inner ring perimeter (Fig 5C). Adhere this ring with hot glue on the inside of ring R1 (Fig 5D); let the glue set until very firm so it does not drop when handling the dome along with the specimen. When in use, this deflector ring will prevent direct, naked light from hitting the specimen directly (Fig 5E & 5F).
11) In order for the dome to sit perfectly flat during use, a small hole or slit must be cut in the base of the dome to allow for the power cord to emerge (Fig 2G & 2H). This is most easily achieved with a hobby knife after the dome is prepared.
12) The complete dome is now ready for use.

Building the mini-ring light for a mini-dome. The process for constructing the mini-ring light largely mirrors that of its larger cousin. A triangle was the chosen solution for the mini-dome to due curvature limitations of LED strip manufacturers; in this design, three short, straight LED strips fit just inside the confines of the mini-dome dimensions.

1. Carefully cut out the mini-dome base template from S2 Appendix.
2. Adhere the LED strips to the template base, using adhesive (Fig 6B & 6C); note that the material used for this base should be heavy, at least 120 lbs/square inch.
3. Note the position of the positive and negative terminals of the LED strips (Fig 6D & 6E). A total of 5 cm of electrical wire (Fig 6D) will be used to jump these LED strips together in series. Strip the insulation off the wire (solid core, point to point wire), and using the same soldering techniques as listed above under steps 3 and 4, solder the positive posts together, then the negative posts (Fig 6F). Note in Fig 6G, two in-series soldering points are completed, while the third is left for the connection of the power cord.
4. Follow the steps above (steps 7–9) for connecting the power cable (R) and the adaptor plug (F), but unlike above, solder the R wires directly to the un-soldered portion of the LED triangle (as in Fig 6G). Once the soldering is complete, the LED ring light should be functional
5. The last step is to mount the mini-LED ring to a microscope slide. This allows the entire unit to be easily moved around the microscope stage using the x-y controls of the microscope itself. This is certainly optional, but strongly recommended. The completed ring light should resemble that which is figured in Fig 6H.

Setup of the mini-dome.

1. The completed dome and LED illumination ring should resemble that pictured in Fig 7A. A cutaway in Fig 7B shows the deflector ring in action, with the specimen in the middle; Fig 7C shows the entire setup fully illuminated.
2. The mini-dome is ideal for imaging whole-mount (e.g. point mount) specimens using the compound microscope; this technique was described originally by [5] using a fiber-optic light source, mylar strips (for diffusion), and metallurgical lenses (non-color corrected). Compound microscope lenses typically have a higher resolving power than zoom lenses, but are also more ‘light-hungry’, and we have found the closer the specimen is to the light source, and hence the more concentrated the light is, the better the illumination for photography. The mini-dome is an ideal solution for this problem, as we are able to situate the LEDs in very close proximity to the subject being photographed, but disperse the light on the inside of the dome. LED lights also do not produce the intense heat of fiber-optic illuminators (whose intensity can produce some rather odd and unsightly off-gassing of illumination domes). Alignment of the dome with the objective lens can be seen in Fig 6D–6F
3. Before constructing the mini-dome, we advise the user to determine the working distance of their lenses for this application. We have found that Plan-APO Leica lenses for metallurgical work (non-color corrected) have a working distance between 10–15mm at 2.5x to 5x; the 10x lens has a major drop-off, and we often have to position the specimen in the illumination dome very close to the oculus itself, lest we run the lens into the dome in trying to achieve focus.
4. Specimens used in the mini-dome are best imaged after being glued to the tip of minuten pins (as summarized in [5]), as these minute pins interfere with the photographic process the least, esp. when compare to typical while-triangular point mounts. Insert the mounted insect into modeling clay to hold the specimen still; as the specimen cannot be manipulated once inside the dome, any fine-tuning of specimen positioning must be completed prior to putting the dome over the specimen and ring light.
5. When printing the mini-dome, the user is directed to print the dome template from S1 Appendix at 30% reduction; this will result in the correct size for the mini-dome light ring.

## Discussion

The dome design provided here was compared to the Leica LED5000 HDI illumination dome, and by all accounts, the dome described here performs comparatively well to the Leica dome. The most obvious design difference between the two domes is the orientation of the LED’s themselves. The dome described here has the LED’s oriented vertically, sending photons directly onto the interior dome surface, and then to the specimen; the Leica dome has some LED’s oriented vertically (as in ours) as well some in the upper portion of the dome, sending light radially throughout the dome. The geometry of the dome described here provides a greater overall dispersion of light onto the specimen being imaged, whereas the Leica dome has the light more diffuse at the center. We feel the Leica dome suffers from the design of the oculus of the dome, where its depth, and the LED’s that surround it, prevent a certain degree of light coming down directly to the specimen. The dome described here has a simple oculus, allowing a direct line of photons to the specimen. In fact, the dome here can have an oculus of any diameter, with a smaller opening rendering more surface for reflecting light.

The use of Styrofoam containers, coupled with fiber optic light sources, as illumination chambers, were published on by [2].). With the publication of [4],), the consistency of Styrofoam containers as light diffusers was called into question, and as a result, we have moved away from Styrofoam and towards domes. The lighting control in a LED dome is without question more constant than the best Styrofoam diffusers coupled with fiber optic lights; for researchers desiring images that are maximally consistent, the LED lighting dome is without a doubt, the superior method. Further, the scalable dome described here would be much more affordable, in fact, than a Styrofoam dome-fiber optic light solution, as research-grade fiber optic illuminators are rather expensive. It should be noted, however, that in the experience of the authors here, raking light effects are more readily accomplished using Styrofoam chambers and fiber optic illuminators.

As mentioned in previous papers on imaging techniques and methods, the format of taxonomic papers, and research in general, has changed very rapidly in the past decade. Journals are no longer limited to black and white line drawing images for taxonomic work, as the cost for color image reproduction is now typically affordable in many journals. On-line image databases continue to grow, and as the critical mass of images for particular groups continues to increase, so too does the power to mine these databases for occurrence, distribution, and general biological data. Together, this brings basic taxonomy into the position it rightfully deserves, at the crux of *all* biological research.

While the construction of digital cameras for scientific photography is certainly beyond the scope of these authors, we have tirelessly tried to improve methods for lighting, as this is one aspect of photography in which we have had some success in improving. The dome system described in [4] was a considerable advancement in this subject, and we feel this research takes the dome one step further in user-friendliness. The LED ring-light described herein largely follows that of [4],), and as LED technology continues to develop at a dizzying pace, the fabrication of domes, up to this point, relied on locating an off-the-shelf source. With the design here described, anyone with the basic equipment found in most entomology labs or departments, can fabricate their own dome, to the precise dimensions they need, for little cost-investment in materials. And the real key element here is size, in that the ratio of specimen size to dome size is critical for excellent photographic results. A large dome illuminating a small specimen is less than ideal, as the reflective surface of the dome is a good distance from the specimen, reducing the intensity of light on the subject, reducing the lens and camera’s ability to resolve fine structures; alternatively, one cannot fit large specimens into a small dome! Using the scalable function of the dome described here, any number of domes can be constructed for the variety of sizes one finds in the course of their research, and figures provide evidence of the variety of insect species that can be imaged using the same dome design, fabricated in different sizes.

The Systematic Entomology Laboratory has used the same LED dome for the past 7 years. While this dome has been a work-horse and provided literally months worth of illumination effort without error, the dome was expensive when compared to the design here. Further, there was a one-size-fits-all mentality in the design; this, in combination with the cost, precluded having different sized domes available for various applications. Additionally, LED design has changed dramatically in the past 7 years, and the LEDs in SEL’s current dome cannot be changed. The design here allows for the flexibility needed by modern taxonomists, and as new LED designs come available, new ring lights can be quickly assembled. In fact, different ring lights, of different wavelengths, can be fabricated as well, extending the limitations even further.

To reiterate, the value of a high-quality color images of species of insects is difficult to place a value on. Much of insect taxonomy is a science based on shape, form, and color. And even as the systematics community continues to develop state-of-the-art molecular-based methods for investigating species, populations, and higher-level phylogenetics, we argue that the need for high-quality images is greater now than ever before. The world of insect taxonomy is extraordinarily rich in beautiful and bizarre forms, all of which can be used to intrigue members of funding agencies and the general public alike. Space agencies world-wide have leveraged funding by providing amazing images of the immediate as well as far off regions of the Cosmos. High-quality images of insects helps to bring people into the amazing Micro-cosmos of planet Earth.

## Supporting Information

S1 Appendix Template for the regular scalable dome system. The size of the completed dome can be adjusted by printing the template at various reduction or enlargement settings using printer preferences (refer to your printer model for directions on how to achieve this). However, this scaling should be conducted in concert with the LED ring gauges. Using a color printer is strongly suggested, as the various colors are referred to in the instructions published here.
(EPS)

S2 Appendix Template for the ‘mini’ scalable dome system. Using a color printer is strongly suggested, as the various colors are referred to in the instructions published here.
(EPS)

S3 Appendix Description of materials, cost, and sources for components of the dome illumination system described herein. (DOCX)
