# Supplementary material for: A Scalable and Modular Dome Illumination System for Scientific Microphotography on a Budget
Source: PLoS One. 2016 May 3;11(5):e0153426. doi: 10.1371/journal.pone.0153426 (PMC4854399; doi:10.1371/journal.pone.0153426)
Supplement: S3 Appendix — (DOCX) [file pone.0153426.s003.docx]

Appendix 3 Description of materials, cost, and sources for components of the dome illumination system described herein.

| **Material** | description | Estimated cost  US Dollar ($) | US suppliers |
| --- | --- | --- | --- |
| **ring light** |  |  |  |
| LED rings | uxcell 2 x 80mm 24 SMD LED Car Angle Eye Halo Ring Light (sizes range from 80 to 100 to 110mm). Color temp: 6000K, white | 7.00-15.00 | [link](http://www.amazon.com/Amico-White-Angel-Light-Headlight/dp/B006W2EKHE/ref=sr_1_cc_1?s=aps&ie=UTF8&qid=1429795367&sr=1-1-catcorr&keywords=led+ring+angel+eye) (80)  [link](http://www.amazon.com/Headlight-Angel-Light-100mm-White/dp/B00LGIXZSE/ref=sr_1_cc_1?s=aps&ie=UTF8&qid=1429795982&sr=1-1-catcorr&keywords=2+x+100mm+Angel+Eyes+SMD+LED+Ring) (100)  [link](http://www.amazon.com/120mm-White-Angel-Headlight-Light/dp/B00LGL0ZSO/ref=sr_1_cc_2?s=aps&ie=UTF8&qid=1429795939&sr=1-2-catcorr&keywords=2+x+120mm+Angel+Eyes+SMD+LED+Ring) (120) |
| solder paste | Rosin Paste Flux #135 in a 2 oz Jar | 10.00 | [link](http://www.amazon.com/Rosin-Paste-Flux-135-Jar/dp/B008ZIV85A/ref=sr_1_6?s=automotive&ie=UTF8&qid=1429730730&sr=1-6&keywords=solder+paste) |
| wire (copper) | EvZ 20m 66ft 20awg Extension Cable Wire Cord for LED Strips Single Colour 3528 5050 | 0.10 | [link](http://www.amazon.com/20awg-Extension-Strips-Single-Colour/dp/B009VCZ4V8/ref=pd_sbs_auto_1?ie=UTF8&refRID=19KA8W88HHE1B9T465NV) |
| 12V wall adaptor | EPtech 12V 2A AC Wall Power Adapter W 5.5mm x 2.5mm DC Cord Plug For IP VoiP Switch PBX | 12.00 | [link](http://www.amazon.com/Power-Adapter-5-5mm-2-5mm-Switch/dp/B00U7J72UK/ref=sr_1_2?s=office-products&ie=UTF8&qid=1429795309&sr=1-2&keywords=12V+wall+adaptor) |
| soldering iron | Neiko 40494A Soldering Iron Kit | 15.65 | [link](http://www.amazon.com/Neiko-40494A-Soldering-Iron-5-Piece/dp/B005HY02FW/ref=sr_1_7?s=automotive&ie=UTF8&qid=1429731102&sr=1-7&keywords=soldering) |
| stick of hot glue | Clear White Hot Melt EVA Glue Adhesive Stick 11mm x180mm | 8.00 | [link](http://www.amazon.com/Pieces-Clear-White-Adhesive-x180mm/dp/B00OKDYPKE/ref=sr_1_21?s=automotive&ie=UTF8&qid=1429733770&sr=1-21&keywords=stick+of+hot+glue) |
| Hot glue gun | Surebonder H-270 High Temperature Full Size Glue Gun | 10.00 | [link](http://www.amazon.com/FPC-H-270-Full-Size-Temperature/dp/B000BQM5CO/ref=sr_1_16?ie=UTF8&qid=1429733869&sr=8-16&keywords=Hot+glue+gun) |
| solder | Kester Pocket Pack Solder 60/40 0.031 0.50 oz. | 6.00 | [link](http://www.amazon.com/Kester-Pocket-Pack-Solder-0-031/dp/B00068IJNQ/ref=sr_1_1?ie=UTF8&qid=1429828136&sr=8-1&keywords=solder) |
